# Spatial and spatio-temporal epidemiological approaches to inform COVID-19 surveillance and control: a review protocol

**DOI:** 10.1186/s13643-022-02016-0

**Published:** 2022-07-14

**Authors:** Julius Nyerere Odhiambo, Carrie B. Dolan

**Affiliations:** 1 Ignite Global Health Research Lab, Global Research Institute, William and Mary, Williamsburg, VA USA; 2 Department of Health Sciences, William and Mary, Williamsburg, VA USA

**Keywords:** Spatial, Spatio-temporal, Geospatial modeling, COVID-19, Africa

## Abstract

**Introduction:**

Severe acute respiratory syndrome coronavirus 2 (SARS-CoV-2) infection that cause coronavirus disease 2019 (COVID-19) have afflicted millions worldwide. Understanding the underlying spatial and temporal dynamics can help orient timely public health policies and optimize the targeting of non-pharmaceutical interventions and vaccines to the most vulnerable populations, particularly in resource-constrained settings. The review systematically summarises important methodological aspects and specificities of spatial approaches applied to COVID-19 in Africa.

**Methods:**

Thematically selected keywords will be used to search for refereed studies in the following electronic databases PubMed, SCOPUS, MEDLINE, CINHAL, and Coronavirus Research Database from January 2020 to February 2022. Two independent reviewers will screen the title, abstracts, and full texts against predefined eligibility criteria based on the study’s characteristics, methodological relevance, and quality. The Preferred Reporting Items for Systematic Reviews and Meta-Analysis (PRISMA) 2020 procedures will be adhered to during the reporting process.

**Discussion:**

COVID-19 modeling remains in its infancy, and research is needed to characterize uncertainty and validate various modeling regimes appropriately. It is anticipated that the review will aid spatial, spatio-temporal modeling decisions necessary for mitigating the current and future pandemics.

**Systematic review registration:**

CRD42021279767

**Supplementary Information:**

The online version contains supplementary material available at 10.1186/s13643-022-02016-0.

## Background

The rapid and devastating spread of the novel COVID-19 pandemic caused by the severe acute respiratory syndrome coronavirus 2 (SARS-CoV-2) pathogen was first discovered in Wuhan, Hubei Province, China, in the latter part of 2019. COVID-19 continues to spread worldwide and has 234 million confirmed cases, and more than 4.8 million deaths have been reported worldwide. In Africa, 9.1 million confirmed cases and 173,480 deaths had been reported as of June 30, 2022 [1]. Its spread has led to a dual health and economic burden resulting in a substantial cost. The International Monetary Fund (IMF) estimated a $ 20 trillion increase in government debt between September 2019 to September 2020 [2]. In mitigating this burden, governments have realigned their healthcare services (cutting down routine testing of other diseases, regulating elective surgeries, and limiting access to non-urgent circumstances) and resources, depriving the most vulnerable populations of essential medical services [3–5].

Although the pandemic continues to overwhelm health systems globally, Africa remains disproportionately affected, with more cases and deaths being reported [6]. The disproportionate spread of COVID-19 across and within countries in Africa has not followed a homogenous pattern. The heterogeneity is attributed to a country’s ability to prevent, detect and mount response strategies [7–9]. This has been exacerbated by incomplete documentation on COVID-19 cases and deaths across the continuum of care [10] due to inefficient and unreliable disease surveillance systems, scarce critical care resources, grossly underfunded and inadequate healthcare facilities, and insufficient training of healthcare workers [11–13]. Additionally, its interaction with poverty-related non-communicable diseases (NCDs) has led to adverse medical outcomes/attributable deaths that cannot be fully quantified [14–16]. Furthermore, the attributed burden remains affected by the unacceptable inequalities in vaccine access and rollout, putting at risk high-risk groups and healthcare workers [12, 17]. Amidst these challenges, concerns about the ability of African countries to achieve the United Nations sustainable development goals (SDGs) continue to be raised [18].

As more transmissible variants continue to spread, driven in part by high human mobility, vaccine hesitancy, low rates of mask utility, supply chain constraints, and distribution inequalities, there is an urgent need of routine surveillance so as to obtain epidemiologically meaningful dynamics at relevant thresholds [13, 19]. Amidst this need, diverse approaches have been employed to unmask the trends and drivers of COVID-19 since its declaration as a global pandemic. However, the utility of spatial and spatio-temporal models has become more relevant and greatly improved the estimation of COVID-19 fine-scale risk [13]. These models have been sustained by the enhanced computational ability creating an ideal environment for identifying and visualizing the regional and global trends of COVID-19 risk. The uptake of these models has contributed to informed and timely public decision-making enabling the optimal allocation of scarce resources, effective control, and containment initiatives, particularly in areas where variants are circulating.

Over the past 2 years, there has been an increase in the literature regarding COVID-19 modeling in space and time [20–23]. However, substantial uncertainty and diverging methodologies of estimation and forecasting have resulted in essential differences in the projections and inference. There have been an ongoing concerns about the availability and quality of the data that have been used to generate critical metrics for quantifying the progress made. This review seeks to appraise the data sources and assess the modeling covariates and methodological rigor of studies employing spatial and spatio-temporal methods. The review can potentially enhance the framework of infectious disease modeling, which is essential for informing modeling decisions for future pandemics in Africa.

## Methods

### Study registration and protocol

This review has been registered with the International Prospective Register of Systematic Reviews (PROSPERO Reference: CRD42021279767). The review will also follow the reporting guidelines outlined in the Preferred Reporting Items for Systematic Reviews and Meta-Analyses 2020 statement (PRISMA 2020) [24] in its procedures to enhance its rigor (Additional file 1).

### Search strategy

**Fig. 1:**
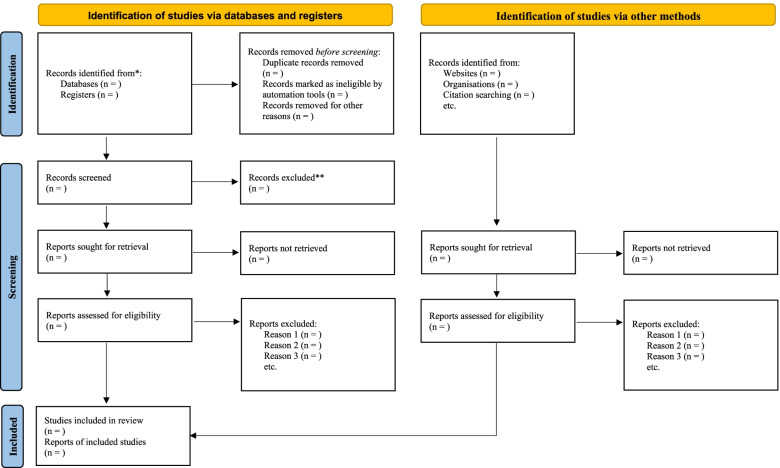
PRISMA 2020 flow diagram

In consultation with a librarian at William & Mary Libraries, a rigorous, phased, and transparent process will be undertaken to search for relevant studies, iteratively select eligible studies, and extract data from the eligible studies. The relevant studies will be searched using the following bibliographic databases, namely PubMed, SCOPUS, MEDLINE via Proquest, CINHAL via EBSCOhost, and Coronavirus Research Database using iterated search terms (Additional file 2). In order to improve the comprehensiveness of the search, thematic keywords will be funneled using Boolean operators, and a combination of medical sub-headings (MeSH) will be used and modified where necessary for each database (Table 1). The snowball technique will be used to manually trace relevant studies in the list of references of the eligible studies up to the point of saturation (i.e., no new information emerged from subsequent articles manually searched). Gray literature will also be searched via Google Scholar, and authors will be contacted for any missing publications. After that, eligible studies will be imported into RefWorks management software. Considering that on January 30, 2020, the World Health Organization officially declared COVID-19 a Public Health Emergency of International Cancer [25], the literature search will be limited to original studies published from January 30, 2020 to February 2022 (Fig. 1).

**Table 1:** Preliminary search string

Theme	Search string
COVID-19	(Betacoronavirus OR Betacoronaviruses), (Corona Virus OR Corona Viruses OR Coronavirus OR Coronaviruses), (COVID OR COVID19 OR Covid-19 ), (CoV OR CoV2 OR HCoV-19 OR nCoV OR 2019nCoV), (Severe Acute Respiratory Syndrome CoV OR severe acute respiratory syndrome coronavirus 2 OR SARS CoV 2 OR SARS-CoV-2 OR SARSCoV OR SARS-CoV OR SARS2)
Model	Spati* OR geospatial OR space-time OR geographic OR mapping OR geospatial OR cluster
Location	Africa

### Inclusion and exclusion criteria

A calibration exercise will ensure that only the correct studies are included and that relevant study information is consistently and accurately captured. This approach improves the methodological rigor and will involve two independent reviewers independently screening the title and abstract of a random sample comprising 5% of the studies. Studies will be eligible for review if they apply one or more of the following spatial or spatio-temporal analyses: exploration (using statistical tools to monitor trends), visualization (cluster analysis to quantify the geographic variation), and modeling (utilizing data to explore COVID-19 risk factors and develop space-time predictive models). We define a spatial model to explicitly incorporate a geographic index for a given area/observations, whereas a temporal model will include a time index (Table 2).

**Table 2:** Study inclusion criteria

Criteria	Description
Study type	Original peer-reviewed journal article utilizing spatio-temporal modeling approaches
Analytical approach	Spatial and spatio-temporal model.
Context	Geography: Africa Language: no language restriction Time frame: all publications from January 30, 2020 to February 2022 will be included.

Studies will be excluded if their abstract or full text is not available. Articles that are not peer-reviewed, such as letters, editorials, comments, book/book sections, conference proceedings, or conceptual papers without findings; studies that did not focus on COVID-19; or did not use GIS or geospatial techniques will also be ineligible for inclusion. Experimental design studies, case series/reports, and studies on the genetic characterization of COVID-19 will also be excluded.

The review team will resolve emerging discrepancies until a consensus is reached. If consensus is not reached, an independent arbitrator will be consulted for possible inclusion or exclusion. The eligibility criteria will be modified if a kappa statistic lower than 50% is observed between the reviewers, indicating low agreement.

### Data abstraction

Using a predesigned online abstraction form, two reviewers will independently and iteratively abstract data on a random sample of 10 articles (i.e., reading the full text of each article and extracting the relevant information). Emerging discordance between the reviewers concerning information extracted will be resolved by consensus among the reviewers and by discussions with a third reviewer. For each eligible study, the following information will be extracted (Table 3):

i. Bibliographic information (author, year, country of origin, spatial and temporal resolution)
ii. Study objective (s) (primary and secondary)
iii. Data sources and modeling covariates
iv. Analytic approach(es) (assumptions, frameworks, cluster detection techniques, model validity test, statistical software)
v. Results and discussions (Findings, study limitations, implications for future research)

**Table 3:** Data abstraction form

1. ***Bibliographic information***	
• Study ID	
• Author, year	
• Country	
• Article title	
• Language study period (start-end)	
• Data source (medical records, multiple sources, others)	
• Type of publication (Journal article, book chapter, grey literature).	
• Data sources	
• Study population/spatial unit (Household, national, province, district facility, census tract, other)	
2. ***Aims***	
Study aims and objectives (primary and secondary)	
3. ***Methodology***	
• Visualization techniques	
• Cluster detection techniques	
• Covariate(s) selection	
• Spatial-temporal modelling approach	
4. ***Results*** (does the paper report data relating to the following?)	
Key findings	
Unexpected results	
5. ***Discussion***	
Key findings	
Unexpected results	
Modeling gap(s)	
Limitations	
6. ***Conclusions and recommendations***	
Modeling issues requiring further attention	
Suggestions for improved analytical approaches for future studies	

### Quality appraisal and risk of bias assessment

The review quality appraisal will be independently done by two reviewers, who will outline the strengths and weaknesses of each study using an adopted tool for assessing quality [26, 27]. This tool will comprise 8-point scoring criteria used to determine the quality of the individual studies based on their study objectives, data source, model validity, results, and conclusions. Four broad categories will, namely: very high (> 13), high (11–13), medium (8–10), and low (< 8), will be used to assess the overall quality level of individual studies. Additionally, screening questions/criteria will guide the scoring process, ranging from 0 (poor) to 2 (good) on each criterion (Additional file 3: Table S1).

### Data synthesis and analysis

This phase will involve three distinct steps: analysis, reporting, and interpreting the review findings. Thematic content analysis will cluster studies based on their spatio-temporal methodological approaches, spatial and temporal resolution, data sources, and modeling approaches to identify the dominant findings and make generalizations. Descriptive summary tables will assess studies based on their geographic location, publication year, data sources, visualization, modeling approaches, and covariates. Results will be reported based on the review objectives, with the practical implications for policy and modeling practices discussed. The current gaps in the spatio-temporal modeling of COVID-19 will also be outlined to guide future research.

## Discussion

Africa’s COVID-19 response strategy has mainly been premised on the World Health Organization (WHO) lead Covax Facility, which set an ambitious target of fully vaccinating 20% of the African population by December, 2021 [28]. To achieve this target, the WHO advocates for local, precise, and targeted distribution of vaccines and non-pharmaceutical interventions based on principled predictions at relevant thresholds. Over the past 2 years, massive research about COVID-19 has been made available shifting and improving our understanding on the control and treatment options available. In particular, data science tools and robust modelling appropriate have been employed to better understand the trajectory of COVID-19 and its attributable factors in space and time. These models have incorporated diverse data sources, covariates and methodology to visualize and describe spatial distributions, identify clusters, and determine patterns of spatial association. Our review will identify knowledge gaps, provide practical insights, help clarify complex and multi-component spatial and spatio-temporal methods, and enhance evidence-based practice and decision-making. Additionally, the review will provide valuable insight to support future bibliographic queries and serve as a resource for outlining the evolution of modeling tools in a major global pandemic. It potentially will offer helpful information specifically on how African countries collect and report data in an era of pandemic health policying.

### Anticipated challenges

We foresee potential challenges relating to our systematic review. First, our search strategy might yield more articles than we might expect. To mitigate this, we will work closely with an information specialist at William & Mary Libraries to ensure that the scope is manageable. Secondly, categorizing and appraising methods accurately and exhaustively might be a challenge. However, we intend to engage with stakeholders with in-depth subject knowledge of COVID-19 modeling to receive feedback important for the review.

## Conclusion

The continuous spread of COVID-19, particularly in unvaccinated populations in Africa, threatens to choke progress made in fighting the pandemic. As high and upper-middle-income countries ease mandates, the risk of global resurgence remains due to the significant disparity in vaccine distribution, particularly in Africa. There is an urgent need to increase equitable access to vaccination and the pace of vaccination. This can be done if policymakers make inferences based on reliable data sources and robust statistical methodology to mitigate the pandemic. By outlining the main methodological approaches used to quantify progress, the results from this review can expand our current understanding of the etiology of COVID-19, orient resource allocation, monitor healthcare access, and plan for effective interventions in Africa and other resource-constrained settings.

## Supplementary Information

**Additional file 1.** PRISMA-P checklist.

**Additional file 2.** PubMed preliminary search results.

**Additional file 3: Table S1**: Risk of bias assessment tool.

## Data Availability

Data sharing does not apply to this article as no datasets were generated or analyzed during the current study.

## Abbreviations

COVID-19: Coronavirus disease 2019
PRISMA: Preferred Reporting Items for Systematic Reviews and Meta-Analyses
PROSPERO: International Prospective Register of Systematic Reviews
SSA: Sub-Saharan Africa
WHO: World Health Organization
