# Supplementary material for: Spatial and spatio-temporal epidemiological approaches to inform COVID-19 surveillance and control: a review protocol
Source: Syst Rev. 2022 Jul 14;11:141. doi: 10.1186/s13643-022-02016-0 (PMC9281235; doi:10.1186/s13643-022-02016-0)
Supplement: Supplementary file 2 — Additional file 2. PubMed preliminary search results. [file 13643_2022_2016_MOESM2_ESM.docx]

**Additional file 2: PubMed preliminary search results**

| **Database** | **Date of Search** | **Keywords** | **Number of publications identified** |
| --- | --- | --- | --- |
| PubMed | 01/28/2022 | ((COVID OR COVID19 OR Covid-19 OR Corona Virus OR Corona Viruses OR Coronavirus OR Coronaviruses OR CoV OR CoV2 OR HCoV-19 OR nCoV OR 2019nCoV OR Severe Acute Respiratory Syndrome CoV OR severe acute respiratory syndrome coronavirus 2 OR SARS CoV 2 OR SARS-CoV-2 OR SARSCoV OR SARS-CoV OR SARS2) AND (Spati* OR geospatial OR space-time OR geographic OR mapping OR geospatial OR cluster)) AND (Africa) | 477 |
| CINHAL via EBCOhost | 02/16/2022 | (covid OR covid19 OR covid-19 OR cov2 OR ncov OR 2019ncov OR coronavirus 2 OR sars-cov-2 ) AND ( ( spati* OR geospatial OR space-time OR geographic OR mapping OR geospatial OR cluster OR model* ) AND africa | 102 |
