# Supplementary material for: Spatial and spatio-temporal epidemiological approaches to inform COVID-19 surveillance and control: a review protocol
Source: Syst Rev. 2022 Jul 14;11:141. doi: 10.1186/s13643-022-02016-0 (PMC9281235; doi:10.1186/s13643-022-02016-0)
Supplement: Supplementary file 3 — Additional file 3: Table S1: Risk of bias assessment tool. [file 13643_2022_2016_MOESM3_ESM.docx]

Supplementary Table 1: Risk of bias assessment tool

|  | **Criterion** | **Considerations** | **Score considerations (0, none, 1, poor, 2, good)** |  |
| --- | --- | --- | --- | --- |
|  | **(A) Screening questions** |  |  | Definition,  Max 4 points |
| 1 | Does the paper clearly address aims and objectives? | Is the paper relevant to the objectives of the systematic review of Bayesian modeling? | 0 not stated  1 stated but vague  2 stated and focussed |  |
| 2 | Is the setting and population clearly defined? | Does the paper clearly state the setting (e.g., number of geographical locations, number of malaria cases)? | 0 not stated  1 stated but vague  2 stated and focussed |  |
|  | **(B) Assessed the validity of the model** | |  |  |
| 3 | Is the model structure clearly described and appropriate for the research question? | Is there a description of model structure (prior for space, time, or space-time)?  Does the model structure include covariates? | 0 not appropriate model structure, or no description of the model  1 incomplete description  2 complete description | Model methods, Max 4 points |
| 4 | Are the modeling methods appropriate for the research question? | Were the modeling methods clearly described and suited to the research question? | 0 not appropriate modeling method, or no description of the method  1 incomplete description  2 complete description |  |
| 5 | Are the parameters, ranges, and data sources specified? | Are all parameters and their ranges reported? | 0 poorly reported  1 some information missing  2 complete reporting of parameters, ranges, and data sources | Model inputs, Max 4 points |
|  |  | Are the data sources for parameters reported? |  |  |
| 6 | Is the quality of data considered? | Are data limitations discussed? | 0 no sources of uncertainty  1 partially addressed, or data inappropriate  2 fully addressed |  |
|  | **(C) Assessed the overall results and study conclusion** | |  |  |
| 7 | Have the results been clearly and completely presented? | Do the results match the aims and objectives? | 0 not reported, very unclear  1 stated, but not directly aligned with the research question  2 valuable and aligned with the research question | Results, Max 4 points |
| 8 | Are the results appropriately interpreted and discussed in context? | Are the results of the study discussed in context and generalisability considered? | 0 no discussion  1 some discussion but key points and/or limitations missed  2 full discussion of key points, limitations discussed |  |
